# Contrast thresholds reveal different visual masking functions in humans and praying mantises

**DOI:** 10.1242/bio.029439

**Published:** 2018-04-15

**Authors:** Ghaith Tarawneh, Vivek Nityananda, Ronny Rosner, Steven Errington, William Herbert, Sandra Arranz-Paraíso, Natalie Busby, Jimmy Tampin, Jenny Read, Ignacio Serrano-Pedraza

**Affiliations:** 1 Institute of Neuroscience, Henry Wellcome Building for Neuroecology, Newcastle University, Framlington Place, Newcastle Upon Tyne, NE2 4HH, UK; 2 Faculty of Psychology, Complutense University of Madrid, Madrid 28223, Spain

**Keywords:** Masking, Motion detection, Praying mantis, Reichardt detector, Visual noise

## Abstract

Recently, we showed a novel property of the Hassenstein–Reichardt detector, namely that insect motion detection can be masked by ‘undetectable’ noise, i.e. visual noise presented at spatial frequencies at which coherently moving gratings do not elicit a response (Tarawneh et al., 2017). That study compared the responses of human and insect motion detectors using different ways of quantifying masking (contrast threshold in humans and masking tuning function in insects). In addition, some adjustments in experimental procedure, such as presenting the stimulus at a short viewing distance, were necessary to elicit a response in insects. These differences offer alternative explanations for the observed difference between human and insect responses to visual motion noise. Here, we report the results of new masking experiments in which we test whether differences in experimental paradigm and stimulus presentation between humans and insects can account for the undetectable noise effect reported earlier. We obtained contrast thresholds at two signal and two noise frequencies in both humans and praying mantises (*Sphodromantis lineola*), and compared contrast threshold differences when noise has the same versus different spatial frequency as the signal. Furthermore, we investigated whether differences in viewing geometry had any qualitative impact on the results. Consistent with our earlier finding, differences in contrast threshold show that visual noise masks much more effectively when presented at signal spatial frequency in humans (compared to a lower or higher spatial frequency), while in insects, noise is roughly equivalently effective when presented at either the signal spatial frequency or lower (compared to a higher spatial frequency). The characteristic difference between human and insect responses was unaffected by correcting for the stimulus distortion caused by short viewing distances in insects. These findings constitute stronger evidence that the undetectable noise effect reported earlier is a genuine difference between human and insect motion processing, and not an artefact caused by differences in experimental paradigms.

## Abstract (precis)

**Summary:** We investigate alternative explanations for a reported difference between human and insect responses to masked moving gratings, and demonstrate that it is a genuine feature of their visual systems.

## INTRODUCTION

The cross-correlation or Elementary Motion Detector (EMD) model was first proposed by Hassenstein and Reichardt to describe the optomotor response of the beetle *Chlorophanus* (Hassenstein and Reichardt, 1956). It has since demonstrated outstanding agreement with behavioural and neurophysiological observations across several forms of motion-elicited behaviour in insects, including tracking (Bahl et al., 2013), collision avoidance (Srinivasan et al., 1991) and landing (Borst and Bahde, 1988). Experiments have elucidated the mechanisms underlying these responses and exposed the neural pathways that mediate detector computations to a remarkable level of detail (Borst, 2014). In humans, the energy model is the standard account of motion perception, being equally successful in explaining behavioural and neurophysiological observations (Adelson and Bergen, 1985). The two models are closely related and (under certain assumptions about their spatial and temporal filters) formally equivalent, suggesting that similar underlying computations can explain visual motion perception from insects to humans.

**Fig. 1.**
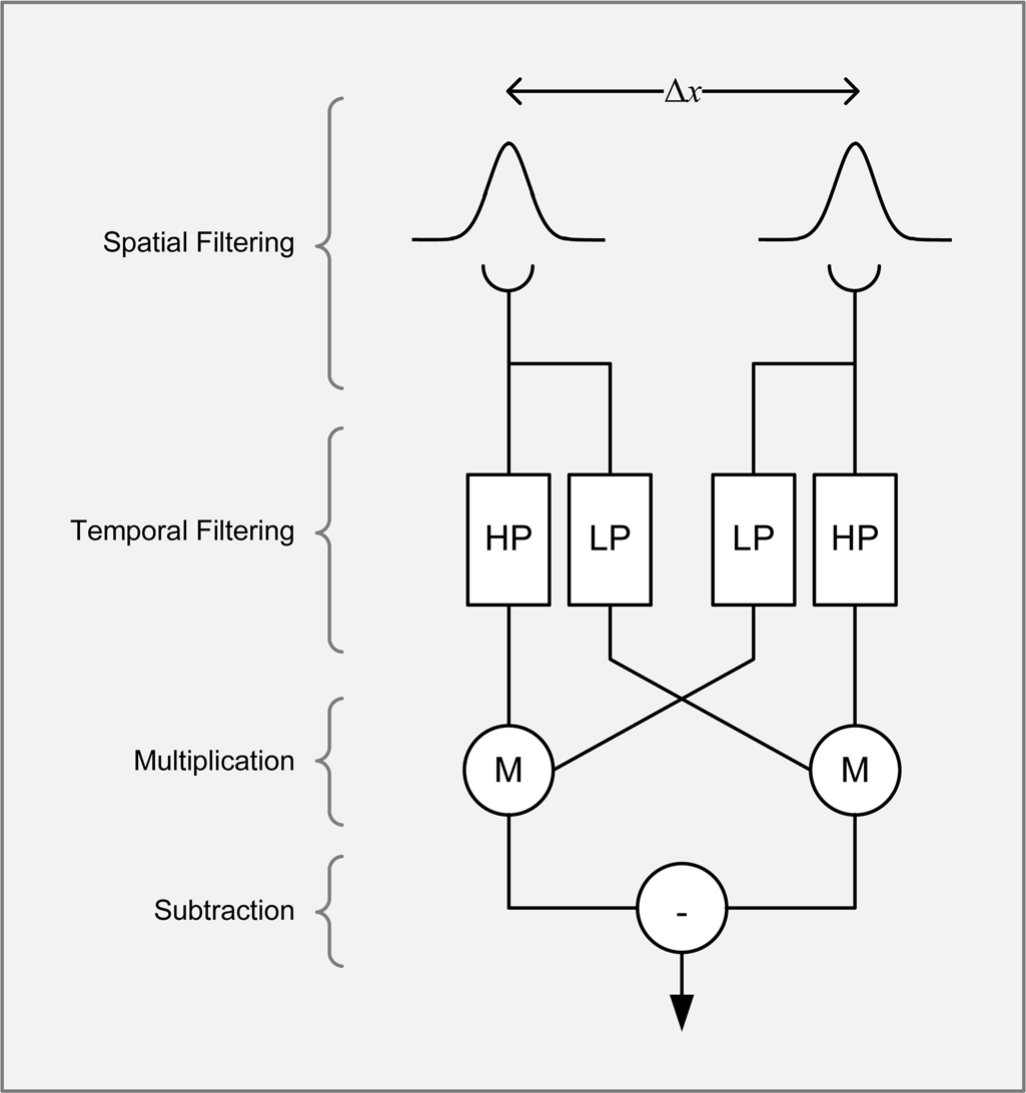
**The Elementary Motion Detector (EMD).** The spatial input from two identical Gaussian filters separated by Δ*x* is passed through high and low pass temporal filters (HP and LP, respectively). The LP output in each subunit is cross-correlated with the HP output from the other subunit using a multiplication stage (M) and the two products are then subtracted to produce a direction-sensitive measure of motion.

The EMD consists of two mirror-symmetrical subunits that compute motion in opposing directions, the outputs of which are combined non-linearly (Fig. 1). We recently showed (Tarawneh et al., 2017) that non-linearity gives EMDs a surprising property, namely that motion detection can be impaired by noise that is undetectable by the animal. In this context, a ‘signal’ is a luminance grating that drifts smoothly in one direction, ‘noise’ is a luminance grating that jumps around with no overall coherent motion (i.e. has a time-varying random phase), and ‘undetectable noise’ is noise at spatial frequencies where signals do not elicit a response [we define ‘detectability’ with respect to observing the behaviour of the animal (Borst, 2014)]. This effect manifests with masked grating stimuli, created by superimposing a noise grating on top of a signal grating and used to measure the animal's ability to detect motion in the presence of noise. In humans, earlier studies (Anderson and Burr, 1985) showed that noise is more effective at masking the signal when its spatial frequency is the same (compared to when it is higher or lower), consistent with the presence of independently operating and frequency-selective motion detection ‘channels’ in the human visual system (Levinson and Sekuler, 1975). Signals are therefore detected by specific channels, and noise spatial frequencies that fall within the active channel's sensitivity band have a stronger masking effect compared to other spatial frequencies. Our previous study (Tarawneh et al., 2017) showed that this is not the case in insects. In remarkable contrast to humans, noise of spatial frequencies much lower than the sensitivity band of insects masks signals nearly as effectively as noise that matches the signal's spatial frequency. For example, an insect may not detect a signal grating at 0.04 cycles per degree (cpd), but noise at 0.04 cpd can nevertheless have a significant impact on the detection rate of a signal grating at 0.2 cpd (nearly as effective as noise at 0.2 cpd). This finding contradicts the intuition that noise outside the frequency sensitivity band of a detector has little influence on its response.

This newly described effect is significant because it highlights a qualitative difference in the response properties of two organisms for which visual motion processing is described by the same model (the EMD). However, confirming the existence of this effect is difficult because published work on masking in insects (Tarawneh et al., 2017) and humans (Anderson and Burr, 1985) has thus far been based on different experimental paradigms and are not therefore directly comparable. In humans, Anderson and Burr (1985) carried out extensive psychometric experiments, measuring contrast thresholds at many different combinations of signal and noise. Their measure of masking was the ratio between the contrast thresholds of masked and unmasked gratings. They used data from two human observers, and contrast thresholds were obtained by the Method of Adjustment (i.e. observers adjusted the contrast of the moving grating by hand until its direction of drift was ‘just discernible’). In mantises, the Method of Adjustment is obviously not feasible. Previously (Nityananda et al., 2015), we have obtained mantis contrast thresholds from psychometric functions using the Method of Constant Stimuli (Lu and Dosher, 2014), where we measured optomotor response rates at several contrasts and then calculated contrast threshold as the contrast corresponding to 50% response rate. This method requires many trials and therefore it was not feasible to investigate response rate at multiple combinations of signal and noise spatial frequencies. Accordingly, we used the masking tuning function as a measure of masking in our 11 mantis observers (Tarawneh et al., 2017). We kept the stimulus contrast fixed, and examined how the response rate varied for noise at different frequencies.

In this paper, we report the results of testing two alternative explanations for the difference in masked grating responses between humans and insects reported in Tarawneh et al. (2017). First, to test whether the difference is due to the use of different masking metrics (contrast threshold versus masking tuning function), we ran masking experiments in both humans and praying mantis (*Sphodromantis lineola*) and quantified masking effects (for both) using contrast thresholds. Second, we repeated mantis experiments with and without applying a correction for the spatial distortion introduced by presenting stimuli on a flat monitor at a short viewing distance. Our results from this independent data set are consistent with the analysis and results reported in Tarawneh et al. (2017). We found that undetectable noise affects insect responses but not the responses of humans, independent of the choice of masking metric or differences in viewing geometry. Our results constitute more conclusive evidence that the undetectable noise effect reported earlier is a genuine difference in visual motion processing between insects and humans, and not an artefact caused by differences in masking metrics or experimental techniques.

## RESULTS

### Experiment H1

We measured contrast detection thresholds of four human observers for direction discrimination in moving gratings under different masking conditions. The signal was a vertical sinusoidal grating (of temporal frequency 8 Hz, spatial frequency *f*_*s*_ and variable contrast) drifting to either left or right in each presentation. We used two different signal spatial frequencies: *f*_*s*_=0.4 cpd and *f*_*s*_=2 cpd. Noise was added in a subset of trials; it had a spatial bandwidth of 1 octave around either 0.4 or 2 cpd and was temporally broadband.

**Fig. 2.**
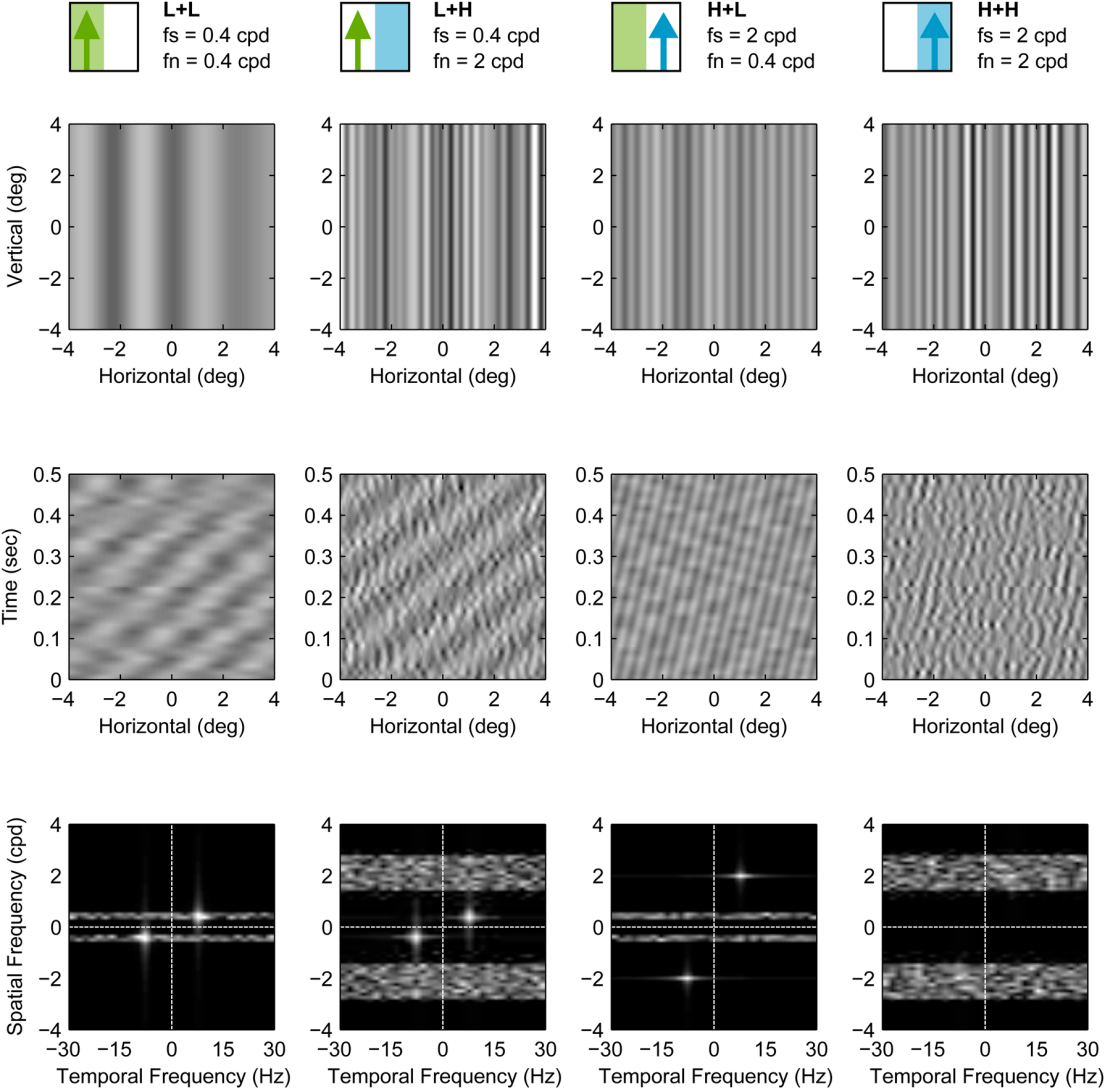
**Masked grating stimulus conditions used in Experiment H1.** Each column represents one stimulus condition. Top row shows still frames of each condition, while middle and bottom rows show corresponding space-time plots and Fourier spatio-temporal amplitude spectra, respectively. In these plots, the signal contrast was set to 0.2. The cartoons at the top represent the conditions graphically and are used in subsequent figures for easy reference (signal is the upwards pointing arrow and noise is the coloured rectangle). The conditions are also labelled using the format Signal+Noise (S+N), where each of S and N is either H or L, indicating high and low spatial frequencies, respectively. For example, L+H indicates low frequency signal and high frequency noise.

We will henceforth refer to the various conditions of our masking experiments using the notation S+N, where S indicates signal frequency and is either H for high frequency (2 cpd) or L for low frequency (0.04 cpd), and N similarly indicates noise frequency. The condition H+L therefore refers to the grating with *f*_*s*_=2 cpd and *f*_*n*_=0.4 cpd. Grating conditions with no noise are simply referred to as H and L. Still frames, space-time plots and the spatiotemporal Fourier amplitude spectra of the masked conditions (L+L, L+H, H+L, H+H) are shown in Fig. 2.

**Fig. 3.**
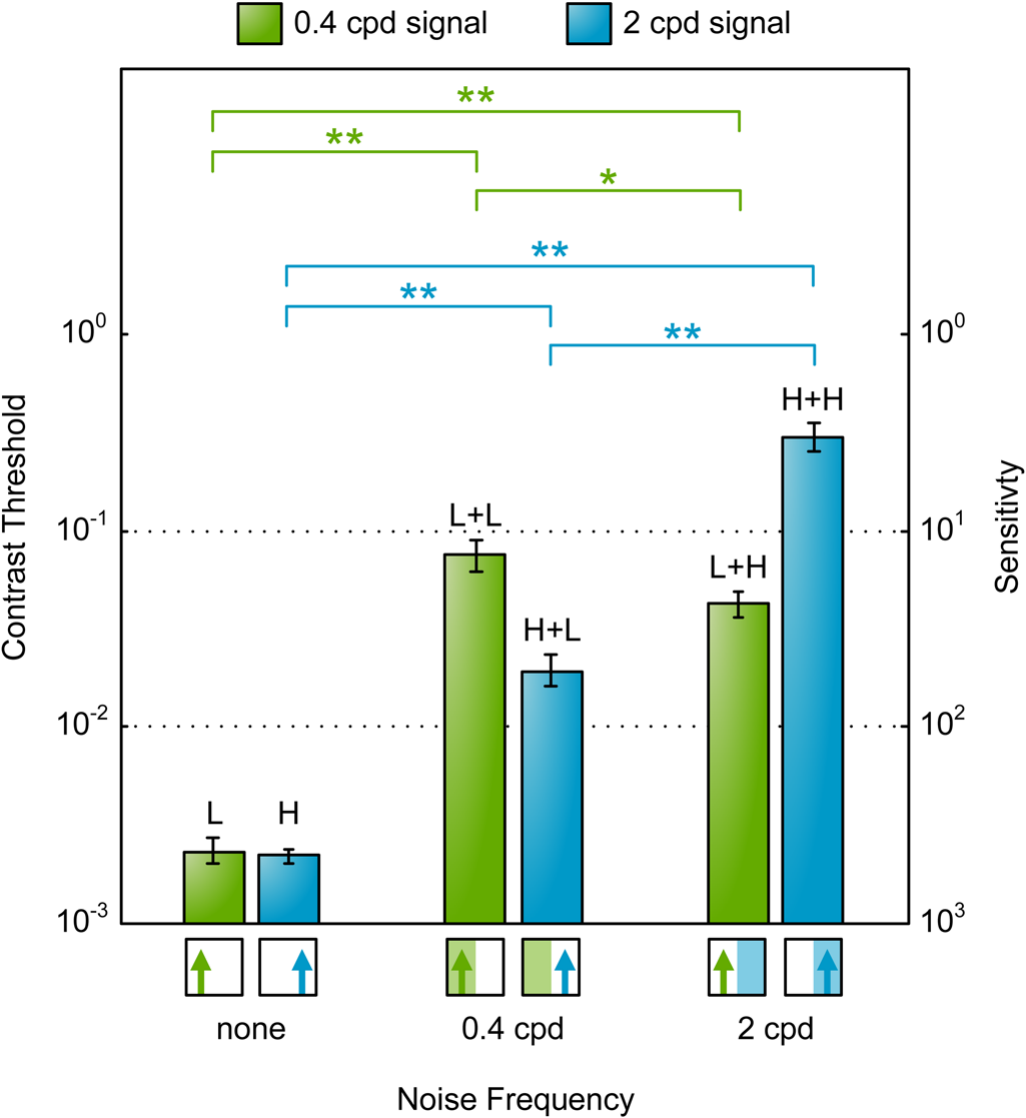
**Human motion detection contrast thresholds for different combinations of signal and noise frequencies (measured in Experiment H1).** Bars show mean contrast detection thresholds (*n*=4) and error bars show ±s.e. of the mean. Horizontal brackets indicate threshold pairs that differ significantly (paired *t*-test, *P≤0.05 and **P≤0.01). Results show that each of the two signals frequencies 0.4 (blue) and 2 cpd (green) was masked significantly higher by same-frequency noise compared to different-frequency noise. Stimuli icons (below bars) and labels (above bars) use the notation introduced in Fig. 2.

Human contrast thresholds are shown in Fig. 3. The thresholds for each signal alone (H and L) do not differ significantly. Adding noise at either frequency caused a significant increase in threshold for both signal frequencies: there were significant differences in thresholds for L+L and L [paired t(3)=13.0, *P*<0.001], for L+H and L [paired t(3)=13.8, *P*<0.001], for H+L and H [paired t(3)=14.7, *P*<0.001] and for H+H and H [paired t(3)=26.0, *P*<0.001]. However, for both the 0.4 and 2 cpd signal frequencies, the threshold was higher when noise and signal frequencies were the same compared to when they were different: there were significant differences in thresholds for L+L and L+H [paired t(3)=4.5, *P*=0.021] and for H+H and H+L [paired t(3)=8.4, *P*=0.003]. These results are consistent with previous studies in human literature which have shown that maximal masking occurs when noise is of equal or near frequency to that of the signal (Anderson and Burr, 1985, 1989).

### Experiment M1

We also ran essentially the same experiment in insects. Mantises were placed in front of a computer screen and viewed full-screen gratings drifting horizontally to either left or right in each trial. The stimuli were the same as described for humans above, except that the spatial frequencies were lowered in order to account for the poorer spatial acuity of insect vision. The high (H) and low (L) spatial frequencies were set to 0.2 and 0.04 cpd, respectively, falling on different sides of the peak spatial sensitivity of the mantis optomotor response (Nityananda et al., 2015). Detection rates were later calculated per condition and individual as the proportion of trials in which the mantis was observed to move in the same direction as the grating.

**Fig. 4.**
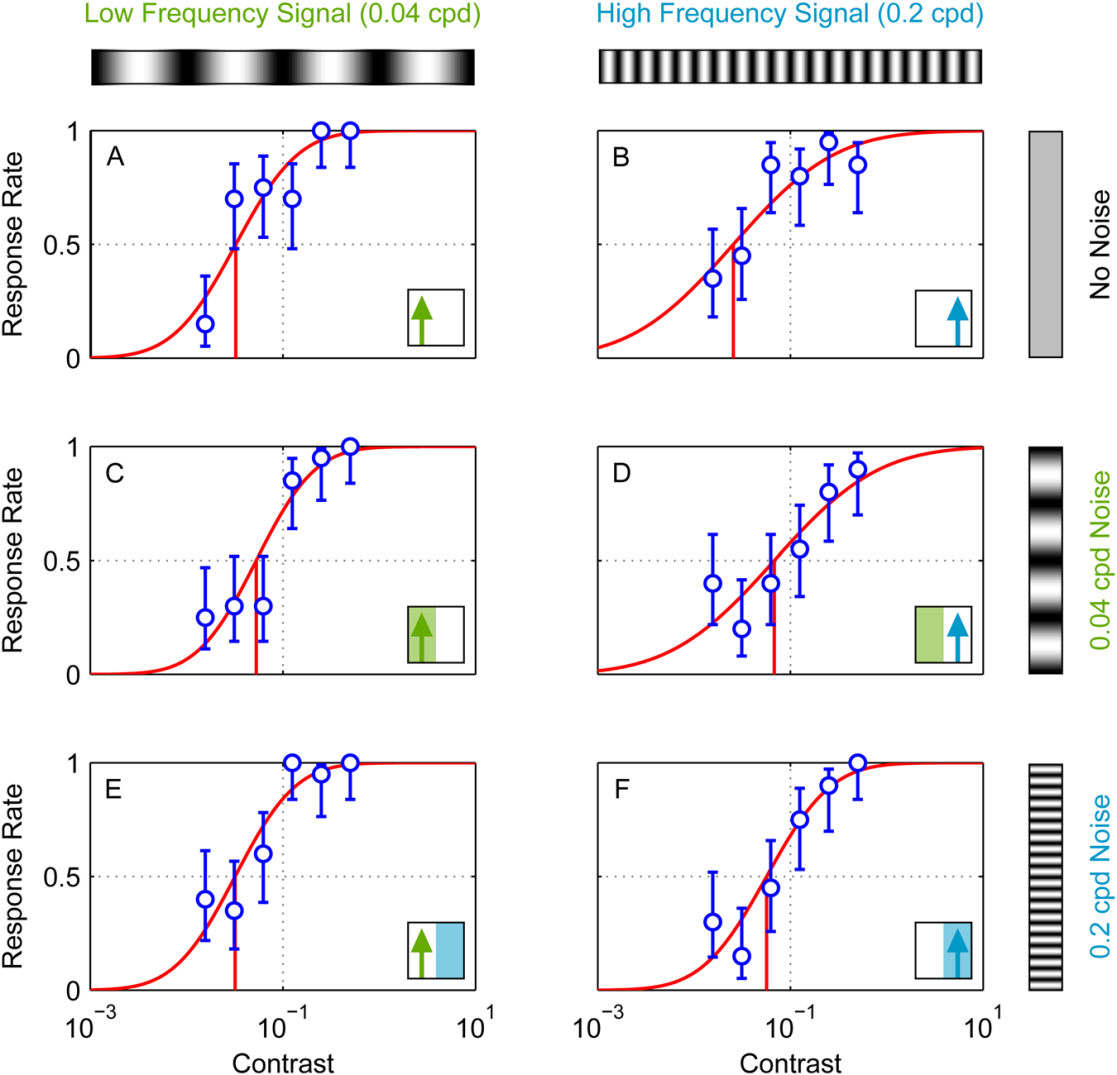
**Responses, fitted psychometric curves and detection thresholds of a single mantis (measured in Experiment M1).** Circles show optomotor response rates (i.e. proportion of trials on which the mantis was coded as moving in the same direction as the signal grating) as a function of signal grating contrast. Error-bars are 95% confidence intervals calculated from simple binomial statistics. Red curves show fitted psychometric function (Eqn 4); red vertical lines mark contrast threshold. (A,C,E) Low-frequency signal (i.e. 0.04 cpd); (B,D,F) high-frequency signal (i.e. 0.2 cpd). Insets at the bottom right corner of each panel indicate signal and noise frequencies as in Fig. 2. (A,B) No noise: stimulus is a pure drifting luminance grating. (C,D) Low-frequency noise, i.e. added to the drifting signal grating is a grating of 0.04 cpd for which phase is updated randomly on every frame. (E,F) High-frequency noise. The data plotted in this figure are all from a single individual (mantis F11) and were measured in Experiment M1.

**Fig. 5.**
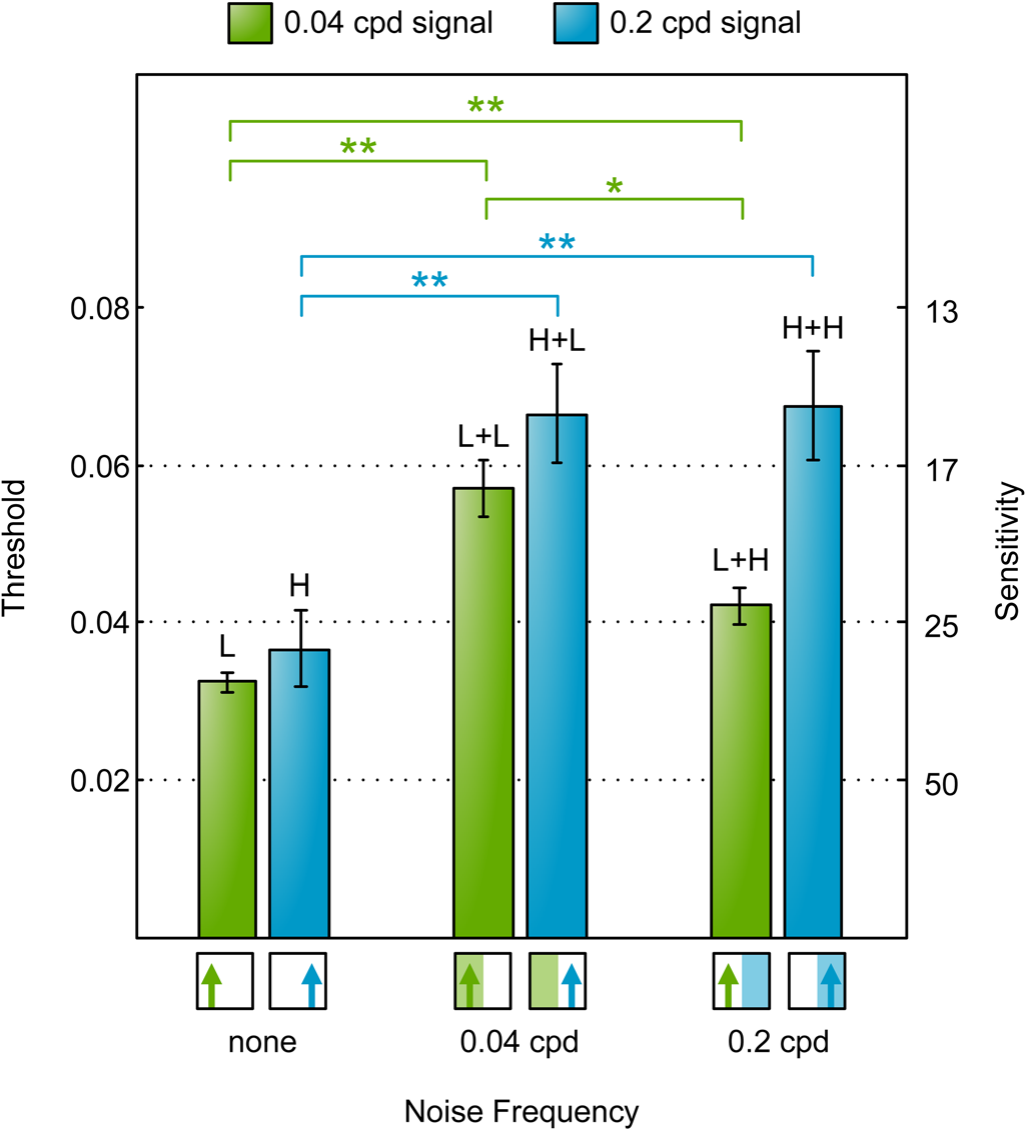
**Mantis motion detection contrast thresholds for different combinations of signal and noise frequencies (measured in Experiment M1).** Bars show mean contrast detection thresholds (*n*=6) and error bars show ±s.e. of the mean. Horizontal brackets indicate threshold pairs that differ significantly (paired *t*-test, *P≤0.05 and **P≤0.01). Stimuli icons (below bars) and labels (above bars) use the notation introduced in Fig. 2. Results show that the 0.2 cpd signal was masked to similar degrees by noise at either frequency, while the 0.04 cpd signal was masked more strongly by the 0.04 cpd noise.

We measured the contrast detection thresholds for each of the conditions L+L, L+H, H+L, H+H as well as the non-masked grating conditions H and L. Fig. 4 shows the response rates and fitted psychometric functions of one individual for illustration. Clearly, adding noise tends to shift the psychometric function rightwards (increasing the contrast thresholds). That is, in the presence of noise, the signal grating has to have higher contrast before it will reliably elicit an optomotor response from the insect. To quantify this we compared the contrast detection thresholds, averaged across six mantises, for the six different conditions (Fig. 5). In the absence of noise, thresholds do not differ significantly between the low and high spatial frequency of the signal gratings [paired t(5)=0.7, *P*=0.494 comparing H and L] as expected, since these two frequencies were chosen to drive the optomotor response equally. Adding low-frequency noise significantly increases thresholds for both signal frequencies: there were significant threshold differences for L+L and L [paired t(5)=7.9, *P*<0.001] and for H+L and H [paired t(5)=4.8, *P*=0.005]. We again see no difference in contrast thresholds depending on the signal frequency [paired t(5)=2.0, *P*=0.096 comparing H+L and L+L]. However, when we add high-frequency noise, we see a very large difference between the two signal frequencies [paired t(5)=6.1, *P*=0.002 comparing H+H and L+H]. High-frequency noise again significantly increases thresholds [paired t(5)=4.0, *P*=0.01 comparing L+H and L, paired t(5)=7.6, *P*<0.001 comparing H+H and H]. The high-frequency signal is affected as badly by high-frequency noise as by low-frequency noise [paired t(5)=0.1, *P*=0.894 comparing H+H and H+L]. However, the low-frequency signal is far less affected by high-frequency noise [paired t(5)=−4.2, *P*=0.009 comparing thresholds for L+H and L+L]. Note that this is not because high-frequency noise has an intrinsically small effect. The high-frequency noise has a very substantial effect on the high-frequency signal, just not on the low-frequency signal.

Experiments H1 and M1 demonstrate the presence of interactions between signal and noise frequencies in both humans and mantises. The responses of the two species, however, were qualitatively different. In humans, noise had a greater effect when presented at the signal frequency and a lesser effect at the other frequency. Mantises, on the other hand, were affected to the same degree by either noise frequency at the 0.2 cpd signal frequency, and more strongly by the noise frequency 0.04 cpd when signal frequency was also 0.04 cpd. In other words, mantises were affected most when noise frequency was equal or lower than signal frequency (across the frequencies 0.04 and 0.2 cpd). This indicates a qualitative difference between the two species.

### Experiment M2

In experiments H1 and M1, the stimuli and experimental procedures were as similar as possible for both humans and mantises, with spatial frequencies chosen appropriate to each species' contrast sensitivity function. However, one difference was that mantises were observing the screen from a much shorter viewing distance (7 cm as opposed to 100 cm for human subjects). When viewing a flat screen from a short distance, the stimulus appears spatially distorted; gratings that are uniform on the screen subtend smaller visual angles at the periphery and may therefore consist of several spatial frequencies (in cpd). Thus, for mantises, the signal gratings effectively varied in spatial frequency across the stimulus, whereas for humans they were much more nearly constant. To test whether this distortion could have influenced our findings from Experiment M1, we repeated the same experiment using a modified stimulus. Previous studies have shown that the optomotor response of the mantis is driven predominantly by the central visual field (Nityananda et al., 2017). The new stimulus was therefore different in three ways: (1) it was limited to the central 85 degrees of the visual field, (2) it was corrected for spatial distortion by introducing a non-linear horizontal transformation, and (3) noise was restricted to a single spatial frequency.

**Fig. 6.**
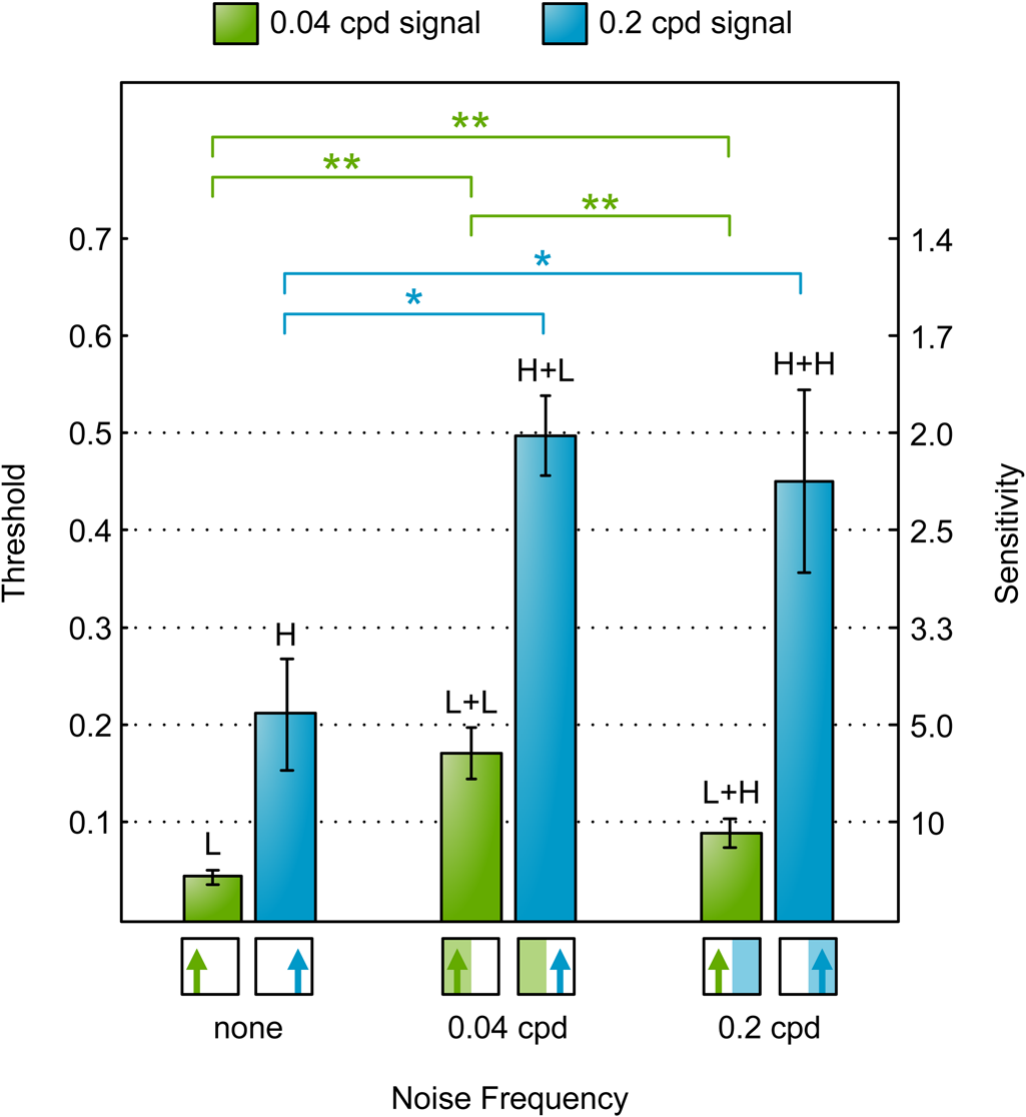
**Mantis motion detection contrast thresholds for different combinations of signal and noise frequencies (measured in Experiment M2).** Bars show mean contrast detection thresholds (*n*=6) and error bars show ±s.e. of the mean. Horizontal brackets indicate threshold pairs that differ significantly (paired-sample *t*-test, *P≤0.05 and **P≤0.01). Stimuli icons (below bars) and labels (above bars) use the notation introduced in Fig. 2. The results show the same qualitative differences observed in Experiment M1 (Fig. 5): the 0.2 cpd signal is masked to similar degrees by noise at either frequency, while the 0.04 cpd signal is masked more strongly by 0.04 cpd noise. This similarity excludes the possibility that mantis and human results were different because stimuli appeared spatially distorted to mantises.

Fig. 6 shows the mean of the contrast detection thresholds, averaged across the six insects, for the six different conditions. Sensitivity was now much lower (the contrast thresholds were higher), particularly for the high frequency, presumably reflecting the alterations to the stimulus. Despite these differences, we found the same qualitative trend observed in Experiment M1. Masking was strongest when noise frequency was equal to or lower than signal frequency of 0.2 cpd. The addition of noise caused a significant increase in thresholds across all conditions: L+L and L [paired t(5)=12.5, *P*<0.001], L+H and L [paired t(5)=8.7, *P*<0.001), H+L and H (paired t(5)=3.8, *P*=0.013] and H+H and H [paired t(5)=2.7, *P*=0.043]. For the 0.04 cpd signal frequency grating, noise at the same frequency caused a significantly larger increase compared to noise at the higher frequency [paired t(5)=6.4, *P*<0.001 comparing L+L and L+H]. There was no significant difference, however, between adding noise at either frequency in case of the 0.2 cpd signal frequency [paired t(5)=1.1, *P*=0.324 comparing H+L and H+H]. That is, noise is equally effective whether added at the signal frequency or at a lower frequency, but less effective when added at a higher frequency. The agreement between our findings in Experiments M1 and M2 suggest that the difference in stimulus viewing distances between humans and mantises, and the resultant spatial distortion, does not explain the qualitative differences in mantis and human responses.

## DISCUSSION

In a previous paper, we documented a striking difference between insect and human visual motion detection (Tarawneh et al., 2017). This difference relates to the robustness of motion discrimination under visual noise (spatial noise that jumps in phase randomly and has no overall coherent motion). In both species, some spatial frequencies are more effective ‘masks’ than others. In human vision, the effectiveness of a given spatial frequency *f*_*n*_ as a mask depends on two factors: (i) sensitivity at *f*_*n*_, and (ii) how close *f*_*n*_ is to signal spatial frequency. Noise at spatial frequencies that are less detectable, or that are further from the signal frequency, is less effective at masking the signal. This is consistent with the widely accepted view that motion detection is mediated by several narrowband channels in the human visual system. Noise is therefore more effective when its spatial frequency is within the sensitivity band of the channel(s) that detect the signal, compared to when it is not. This apparently obvious result does not hold in insects, however, despite the fact that motion detection in both insects and humans is explained equally well by the same model. We found previously that noise of spatial frequencies much lower than the signal is nearly just as effective in masking the signal (compared to noise of the same frequency). This is true even when noise is at such low frequencies that signals elicit no response.

In our previous work (Tarawneh et al., 2017), we predicted this effect by deriving formulae for an EMD's response to stimuli composed of multiple gratings. Briefly, the reason why undetectable noise affects insects but not humans is as follows. When an EMD is presented with a stimulus consisting of signal and noise gratings, it produces a sum of independent responses to signal and noise, plus a non-linear interaction term between signal and noise. It is the latter that causes the undetectable noise effect; an interaction between signal and noise gratings can introduce a response term dependent on the parameters of both. Therefore, a noise grating that is undetectable when presented independently (due to its low spatial frequency) can ‘pair’ with a detectable signal grating at the non-linear multiplication stage (Fig. 1) and influence the EMD's output. In humans, the bandpass spatial filters attenuate interaction terms at the forefront so signal motion detection is unaffected by the addition of independently undetectable noise (Anderson and Burr, 1987). Insects, however, have low-pass spatial filters and rely on the subtraction stage of the model (Fig. 1) to eliminate low spatial frequency responses. Interaction terms (in insects) can therefore survive both spatial filtration and the subtraction stage, causing them to contribute to the EMD's output and introduce the undetectable noise effect we described.

Because this prediction is so counter-intuitive, it was important to validate it experimentally. In our previous study, we tested how masking affects praying mantises by measuring the relative drop in response rate (masking tuning function) for a stimulus with a fixed signal contrast. Although the results were consistent with our prediction, they were not directly comparable to published results in humans that relied on a different masking metric (contrast threshold elevation). Additionally, some experimental adjustments were necessary to be able to measure mantis responses, such as using large-field stimuli so as to trigger the optomotor response. The question therefore remains of whether any observed differences are caused by actual differences in motion processing between humans and insects, or by differences in experimental design.

This paper presents the results of masking experiments in which we used the same experimental paradigm in humans and insects, and reduced experimental differences as much as possible. Here, we selected two spatial frequencies, one high and one low, on either side of the organism's peak sensitivity. These were chosen to be equally detectable, or more precisely, to have equal contrast thresholds on a motion direction discrimination task. We then examined the effect of adding noise either at the same frequency, or the other frequency. We measured thresholds using the psychometric function obtained with the Method of Constant Stimuli, with the contrast of the signal grating as the varying parameter (Fig. 4). Additionally, we tested whether differences in viewing geometry (specifically, the warping of spatial frequencies due to short viewing distances) had any (qualitative) impact on the results.

Our findings were as follows. In humans, noise had a significantly stronger masking effect when added at the same frequency as the signal; noise at a higher or lower frequency had less effect (Fig. 3). In mantises, noise was equally effective whether added at the signal frequency or at a lower frequency, but less effective when added at a higher frequency (Fig. 6). Applying a correction for the horizontal distortion introduced by viewing a large field stimulus on a flat screen had no qualitative effect on this result. In summary, these findings agree with our previous results using response rates at a fixed contrast (i.e. masking tuning function) and are consistent with our earlier prediction: the undetectable noise effect is a genuine property of the current model of insect motion perception (Tarawneh et al., 2017).

## MATERIALS AND METHODS

### Human Experiment H1

#### Subjects

Data in Experiment H1 were collected from four subjects, all with experience in psychophysical experiments. Two were authors and two were naïve to the purposes of the study. Ethical approval was obtained from the Faculty of Psychology at Complutense University of Madrid, Spain. Informed consent was obtained from all participants.

#### Visual stimulus

The stimulus had signal and noise vertical sinusoidal gratings. The signal moved either leftwards or rightwards on each trial while noise had no net coherent motion. Signal gratings had a temporal frequency of 8 Hz and a spatial frequency of either 0.4 or 2 cpd. Pilot work indicated that these spatial frequencies were on either side of peak sensitivity and that thresholds were similar for both. Noise gratings were Gaussian noise (in the luminance domain) that was filtered using an ideal band-pass filter with a bandwidth of 1 octave, around a frequency *f*_*n*_, and a power spectral density of 0.02 (cpd)^−1^. On each trial, the noise grating was either (1) not present, (2) added with *f*_*n*_=0.4 cpd, or (3) added with *f*_*n*_=2 cpd. The phase spectrum of the noise was updated randomly on every Cathode Ray Tube (CRT) frame (refresh rate was 60 Hz), making it temporally broadband up to the Nyquist temporal frequency of 30 Hz. The contrast levels of the signal and noise components were summed at each pixel (parameters were chosen to ensure that no clipping occurred). Each presentation lasted for 1 s. Still frames, space-time plots and spatiotemporal Fourier amplitude spectra of the masked condition stimuli used in Experiment H1 are shown in Fig. 2.

#### Experimental setup

Participants viewed stimuli on a 19″ Eizo T765 CRT monitor from a distance of 100 cm. The monitor had a resolution of 1280×1024 pixels, 14-bit luminance levels and subtended a visual angle of 19.18×15.37 degrees at the viewing distance of participants. Its mean luminance was 57 cd/m^2^. Luminance was gamma corrected (gamma=2.31) using a Minolta LS-100 (Konica Minolta, Tokyo, Japan). A chin-rest (UHCOTech HeadSpot, University of Houston College of Optometry, Houston, USA) was used to stabilise the subject's head. Experiments were administered by a Matlab script using Psychophysics Toolbox Version 3 (PTB-3) (Brainard, 1997; Pelli and Brainard, 1997; Kleiner et al., 2007). DataPixx Lite and ResponsePixx Handheld devices (VPixx Technologies, Saint-Bruno, Canada) were used to render stimuli and capture participant responses.

#### Experimental procedure

Subjects indicated perceived direction of motion (left or right) after each presentation by button presses and their contrast thresholds (for a performance of 82% correct) were calculated using adaptive Bayesian staircases (Treutwein, 1995). The basic characteristics of the staircases were: (a) uniform prior distribution (Emerson, 1986) with a starting Michelson contrast of 0.1; (b) the likelihood function used was the logistic function (see Eqn 1 in Chacón et al., 2015), with spread value of 0.8, delta parameter of 0.01, lapsing rate of 0.01 and guessing rate of 0.5; (c) the value of the contrast in each trial was obtained from the mean of the posterior probability distribution (King-Smith et al., 1994); (d) the contrast threshold was estimated from the mean of the final probability density function; (e) the staircase ended after a fixed number of trials (Anderson, 2003), in particular each staircase consisted of 50 trials and contrast thresholds were averaged across three staircase repeats per condition for each subject.

### Mantis Experiments M1, M2

#### Insects

The insects used in experiments were six adult females of the species *Sphodromantis lineola*. Each insect was stored in a plastic box of dimensions 17×17×19 cm with a porous lid for ventilation and fed a live cricket twice per week. The boxes were kept at a temperature of 25°C and were cleaned and misted with water twice per week.

#### Visual stimulus – Experiment M1

**Fig. 7.**
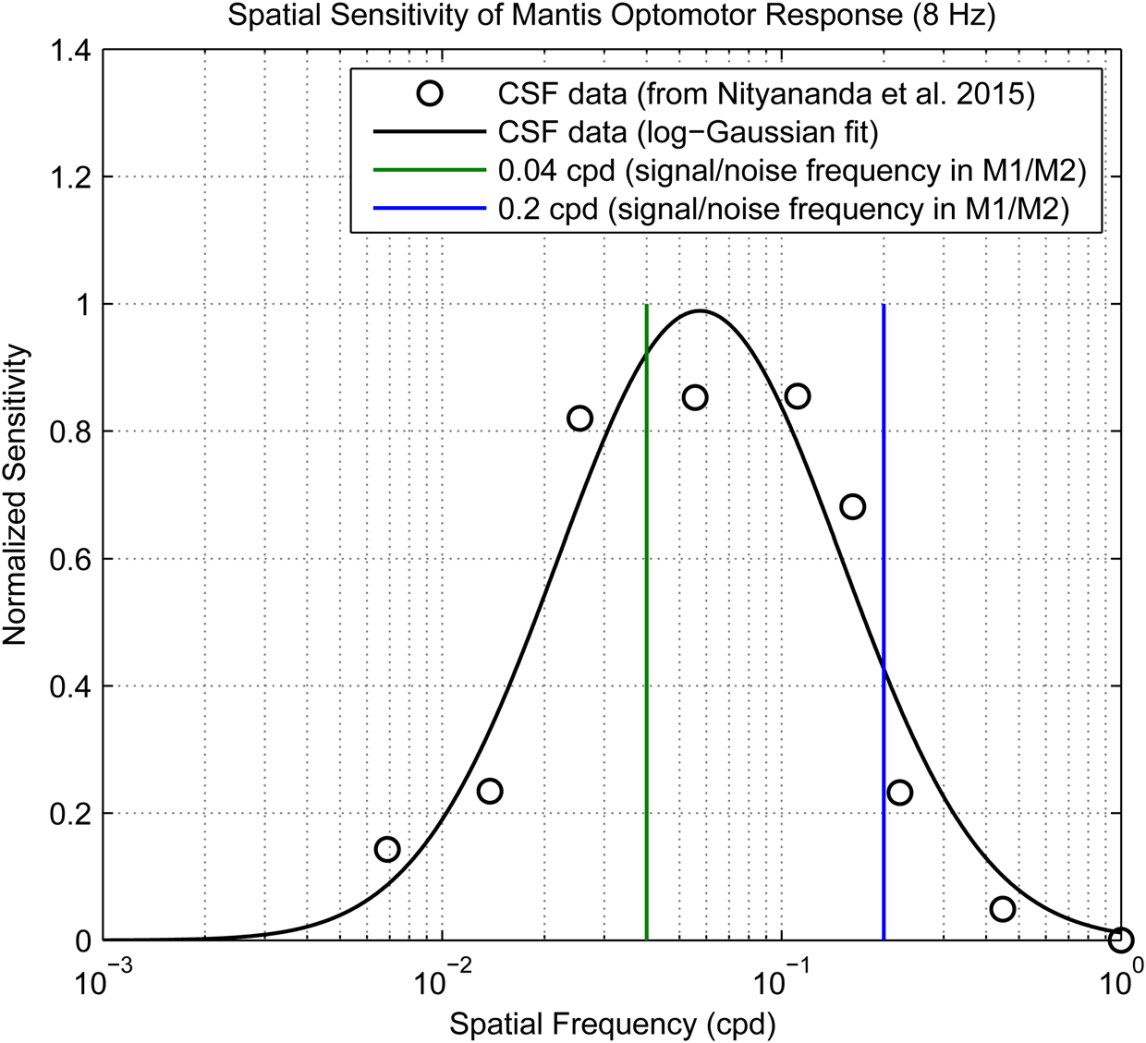
**The spatial contrast sensitivity function of mantis optomotor response.** The spatial frequencies used for signal and noise in experiments M1/M2 (0.04 and 0.2 cpd) are indicated on the plot using green/blue vertical lines. Contrast sensitivity data points are from Nityananda et al. (2015) and were corrected to adopt the same notation for converting between pixels and visual degrees as in Experiment M1 [i.e. averaging over screen width, instead of a single spatial period as in Nityananda et al. (2015)].

In Experiment M1, signal spatial frequencies (*f*_*s*_) were either 0.04 cpd or 0.2 cpd and noise was either (1) not present, (2) added with *f*_*n*_=0.04 cpd, or (3) added with *f*_*n*_=0.2 cpd. The frequencies 0.04 and 0.2 cpd were chosen to be on different sides of the mantis optomotor contrast sensitivity function (Fig. 7). Noise again had a spatial bandwidth of 1 octave. For each of the six combinations of grating frequency and noise setting, trials were run with grating Michelson contrast levels of [2^−6^, 2^−5^…2^−1^] to calculate contrast detection thresholds. There were thus 36 different conditions in total. A total of six mantises each ran 10 repeats of each condition (360 trials).

#### Visual stimulus – Experiment M2

In this experiment we measured contrast thresholds using masked grating stimuli which have been corrected for horizontal distortion. A grating rendered on a flat screen for which spatial periods are constant in pixels is non-uniform in visual degrees (Anderson and Burr, 1987): a given number of pixels at the edge of the screen projects to a smaller angle than the same number directly in front of the viewer. This distortion is generally neglected in human psychophysics but is potentially important at the small viewing distance (7 cm) used in our experiments. To correct for this, we applied a non-linear horizontal transformation in this experiment so that grating periods subtend the same visual angle irrespective of their position on the screen, using the technique described in Nityananda et al. (2017). This was achieved by calculating the visual angle corresponding to each screen pixel using the function:(1)
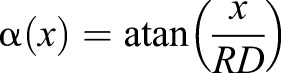
where *x* is the horizontal pixel position relative to the centre of the screen, α(*x*) is its visual angle, *R* is the horizontal screen resolution in pixels/cm and *D* is the viewing distance. To an observer standing more than *D* cm away from the screen, a grating rendered with this transformation looked more compressed at the centre of the screen compared to the periphery. At *D* cm away from the screen, however, grating periods in all viewing directions subtended the same visual angle and the stimulus thus appeared uniform (in degrees) as if rendered on a cylindrical drum. This correction only works perfectly if the mantis head is positioned at the horizontal centre of the screen. As an additional precaution against spatial distortion or any stimulus artefacts caused by oblique viewing we restricted all gratings to the central 85° of the visual field by multiplying the stimulus luminance levels *L*(*x*, *y*, *t*) with the following Butterworth window:(2)
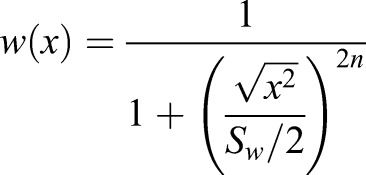
where *x* is horizontal pixel position relative the middle of the screen, *S*_*w*_ is window size (distance between the 0.5 gain points) in pixels, chosen as 512 pixels in our experiment (subtending a visual angle of 85° at the viewing distance of the mantis) and *n* is window order (chosen as 10). This restriction minimised any spread in spatial frequency at the mantis retina due to small deviations in mantis head position during trials or imperfections in our correction formula described by Eqn 1.

With the above manipulations the presented stimulus (as a function of pixel horizontal position *x* and frame number *j*) was:(3)
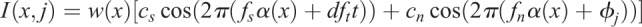
where *I* is pixel luminance, normalised to the range [0, 1], where 0 and 1 are the lower/upper bounds on screen luminance (0.161 and 103 cd/m^2^ respectively), *c*_*s*_ is signal contrast (varied across trials), *c*_*n*_ is noise contrast (fixed at 0.2), *f*_*s*_ and *f*_*n*_ are the spatial frequency of signal and noise respectively (both varied across trials), *f*_*t*_ is signal temporal frequency (fixed at 8 Hz), *d* indicates motion direction (either 1 or –1 on each trial), *φ*_*j*_ is picked randomly from a uniform distribution between 0 and 1 on every frame of the trial, *t* is time in seconds (given by *t*=*j*/85), and *α*(*p*) is the pixel visual angle according to Eqn 1.

**Fig. 8.**
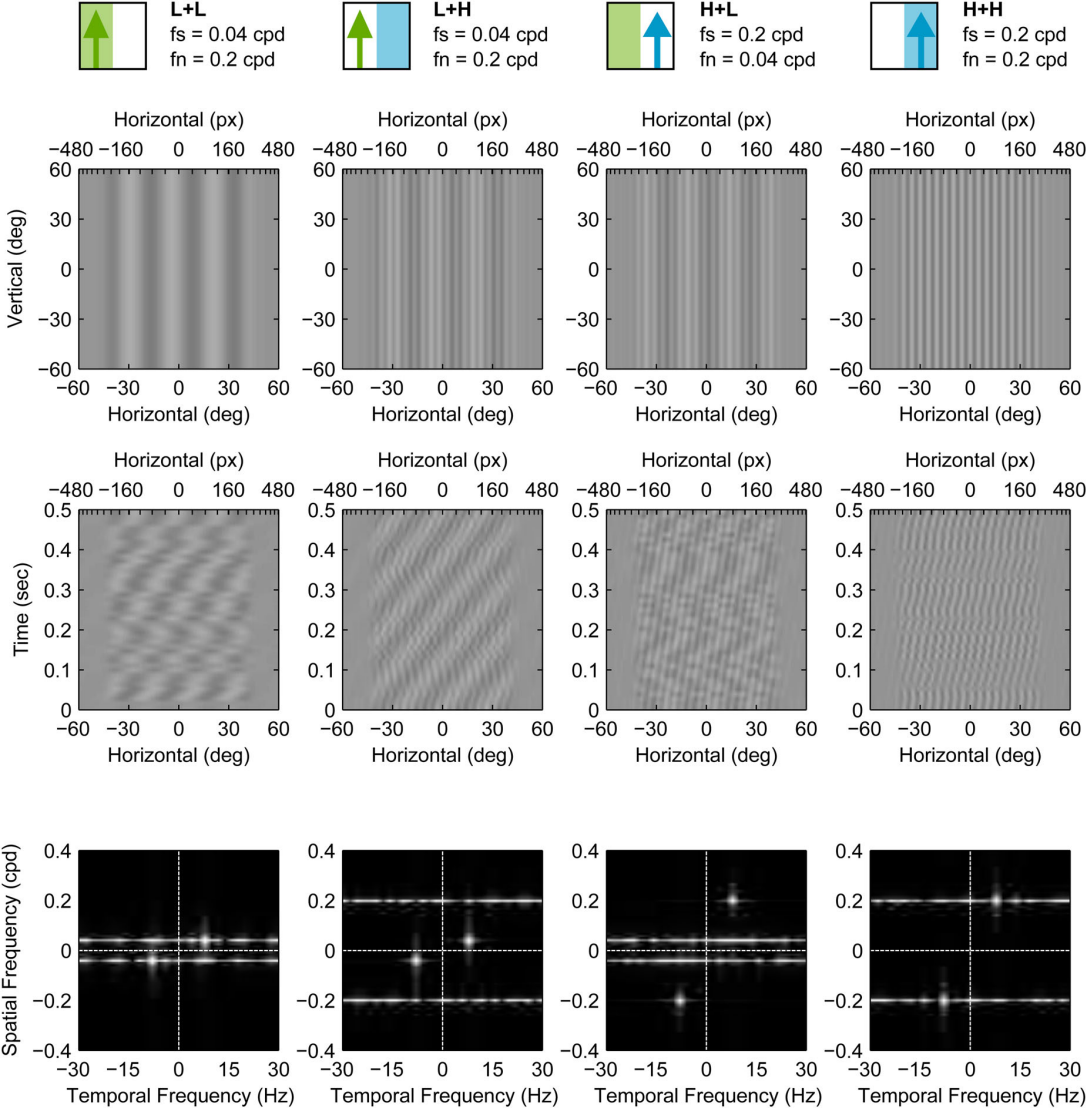
**Masked grating stimulus conditions used in Experiment M2.** Each column represents one stimulus condition. Top row shows still frames of each condition, while middle and bottom rows show corresponding space-time plots and Fourier spatio-temporal amplitude spectra, respectively. In these plots the signal contrast was set to 0.1. These stimuli conditions are similar to their correspondents in Experiment H1 (Fig. 2) but were modified in three ways: (1) they were limited to the central 85 degrees of the visual field, (2) they were corrected for spatial distortion by introducing a non-linear horizontal transformation, and (3) their noise was restricted to a single spatial frequency. Stimuli icons and labels (top row) use the notation introduced in Fig. 2.

Still frames, space-time plots and spatiotemporal Fourier amplitude spectra of the masked condition stimuli used in Experiment M2 are shown in Fig. 8.

#### Experimental setup

The setup consisted of a CRT monitor (HP P1130, Hewlett Packard, Palo Alto, USA) and a 5×5 cm Perspex base onto which mantises were placed hanging upside down facing the (horizontal and vertical) middle point of the screen at a distance of 7 cm. The Perspex base was held in place by a clamp attached to a retort stand and a web camera (USB B3 HD Webcam, Kinobo, London, UK) was placed underneath, providing a view of the mantis but not the screen. The monitor, Perspex base and camera were all placed inside a wooden enclosure to isolate the mantis from distractions and maintain consistent dark ambient lighting during experiments.

The screen had physical dimensions of 40.4×30.2 cm and pixel dimensions of 1600×1200 pixels. At the viewing distance of the mantis the horizontal extent of the monitor subtended a visual angle of 142°. The mean luminance of the monitor was 51.6 cd/m^2^ and its refresh rate was 85 Hz.

The monitor was connected to an OptiPlex 9010 (Dell, Round Rock, USA) computer with a Quadro K600 graphics card (Nvidia, Santa Clara, USA) and running Microsoft Windows 7. All experiments were administered by a Matlab 2012b (MathWorks) script which was initiated at the beginning of each experiment and subsequently controlled the presentation of stimuli and the storage of keyed-in observer responses. The web camera was connected and viewed by the observer on another computer to reduce the processing load on the rendering computer's graphics card and minimise the chance of frame drops. Stimuli were rendered using Psychophysics Toolbox Version 3 (PTB-3) (Brainard, 1997; Pelli and Brainard, 1997; Kleiner et al., 2007).

#### Experimental procedure

Each experiment consisted of a number of trials in which an individual mantis was presented with moving gratings of varying parameters. An observer viewed the mantis through a camera underneath while blind to the stimulus and was asked to classify the observed optomotor response of the insect (if any) in each trial as (1) moved left, (2) moved right or (3) did not move. There were equal repeats of left-moving and right-moving gratings of each condition in all experiments. Trials were randomly interleaved by the computer.

In between trials a special ‘alignment stimulus’ was presented and used to steer the mantis back to its initial body and head posture as closely as possible. The alignment stimulus consisted of a chequer-like pattern which could be moved in either horizontal direction by keyboard shortcuts and served to re-align the mantis by triggering the optomotor response.

#### Calculating contrast detection thresholds

After conducting Experiments M1 and M2 we calculated motion probability *P* (for each individual and stimulus condition) as the proportion of trials in which the mantis was observed to move in the same direction as the signal grating. As in Nityananda et al. (2015), the number of trials on which the mantis was coded as moving in the opposite direction was negligible. We then fitted the individuals' responses using the psychometric function:(4)
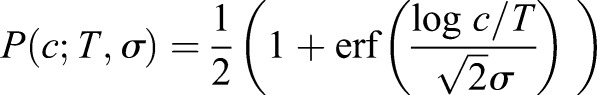
where *c* is the contrast of the signal grating, *T* is the contrast detection threshold (the contrast corresponding to *P*=0.5) and *σ* represents the function's steepness. This function assumes zero baseline response since, unlike humans, mantids could not be have been forced to guess stimulus direction and their coded responses were ternary (moved left, moved right or did not move). For the same reason, motion probability *P* was used instead of the metric ‘percent correct’ that is more commonly used in forced choice tasks (Treutwein, 1995). Also note that this psychometric function predicts observed responses and is therefore a product of mantis and observer sensitivities.

Assuming mantis responses had a simple binomial distribution, we used maximum likelihood estimation (Watson, 1979) to calculate the psychometric function parameters, i.e.(5)
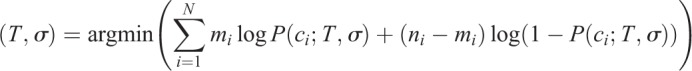
where the subscript *i* indicates different contrast levels, *n*_*i*_ is the total number of trials done for contrast *c*_*i*_, *m*_*i*_ is the number of trials in which the mantis moved in the grating direction, *P* is motion probability given by Eqn 4 and *N* is the number of contrast levels. Contrast detection thresholds were estimated for each insect's individual data, and detection thresholds were then averaged across the population.

#### Data collection

In Experiment M1, six animals ran two blocks of trials each. Each block had 360 randomly interleaved trials consisting of 60 repeats of each grating condition and mantis responses were coded by WH.

In Experiment M2, six animals ran a single block of trials each. Each block had 360 randomly interleaved trials consisting of 60 repeats of each grating condition and mantis responses were coded by SE.
